# Bis{[2,2′-(5,8,11-tri­thia-2,14-di­aza­penta­deca-1,14-diene-1,15-di­yl)diphenolato]palladium(II)} aceto­nitrile monosolvate

**DOI:** 10.1107/S1600536813014712

**Published:** 2013-06-08

**Authors:** Louise Nicole Dawe, Bibhutosh Adhikary, Julie L. Collins, C. Robert Lucas

**Affiliations:** a Department of Chemistry, Memorial University of Newfoundland, St John’s, NL, A1B 3X7, Canada; b C-CART X-Ray Diffraction Lab, Memorial University of Newfoundland, St John’s, NL, A1B 3X7, Canada

## Abstract

The asymmetric unit of the title compound, [Pd(C_22_H_26_N_2_O_2_S_3_)]_2_·CH_3_CN, contains two complex mol­ecules and a single uncoordinated lattice aceto­nitrile solvent mol­ecule. The Pd^II^ cations have a *trans*-N_2_O_2_ square-planar geometry and the superposition of the two crystallographically independent Pd^II^ complexes yields an overall r.m.s. deviation of 0.292 Å. The Pd⋯Pd separation in the asymmetric unit is 3.3776 (3) Å, while the PdN_2_O_2_ plane–plane fold angle is 1.62 (7)°. A short inter­molecular S⋯S contact between the central S atom of one complex and its inversion-related symmetry equivalent of 3.663 (2) Å is observed. Part of the ligand chain (S—C—C—S) in each complex mol­ecule is disordered over two orientations and refined occupancies that converged to 0.450 (10) and 0.550 (10) for the one complex mol­ecule, and 0.789 (9) and 0.211 (9) for the other.

## Related literature

For the synthesis of the ligand 5,8,11-tri­thia-2,14-di­aza­penta­deca-1,14-diene-1,15-di­yl)diphenolate, and the related com­plexes [2,2′-(5,8-di­thia-2,11-diazo­dodeca-1,11-diene-1,12-di­yl)diphenolato]cobalt tetra­fluoro­borate and [2,2′-(5,8-di­thia-2,11-diazo­dodeca-1,11-diene-1,12-di­yl)diphenolato]nickel acetate, see: Lucas *et al.* (2011*a* ▶). For the preparation of the starting material, bis­(aceto­nitrile)­dichloro­palladium(II), from which the title complex was synthesized, see: Mathews *et al.* (2003 ▶). For a copper complex containing the same ligand as the title complex, bis­[μ_2_-2,2′-(5,8,11-tri­thia-2,14-di­aza­penta­deca-1,14-diene-1,15-di­yl)diphenolato]dicopper(II), see: Lucas *et al.* (2011*b* ▶). Lucas *et al.* (2011*b* ▶) also reports the related [2,2′-(5,8-di­thia-2,11-di­aza­dodeca-1,11-diene-1,12-di­yl)diphenolato]copper(II). For Pd catalysts containing salicylaldimine (sal) ligands, see: Jin *et al.* (2010 ▶). For a discussion on the coordination capabilities of Pd^II^, see: Aullón & Alvarez (1996 ▶). For a description of the Cambridge Crystallographic Database, see: Allen (2002 ▶).
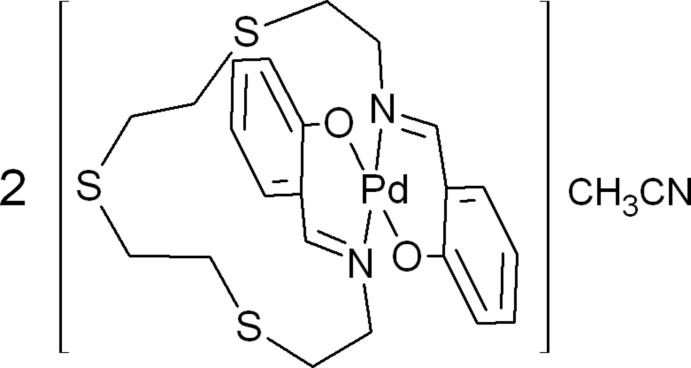


## Experimental

### 

#### Crystal data

[Pd(C_22_H_26_N_2_O_2_S_3_)]_2_·C_2_H_3_N
*M* *_r_* = 1147.11
Monoclinic, 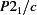

*a* = 14.7232 (5) Å
*b* = 16.2151 (5) Å
*c* = 20.9433 (8) Å
β = 106.087 (1)°
*V* = 4804.2 (3) Å^3^
*Z* = 4
Mo *K*α radiation
μ = 1.06 mm^−1^
*T* = 153 K
0.39 × 0.39 × 0.24 mm

#### Data collection

Rigaku Saturn70 diffractometer
Absorption correction: numerical (*ABSCOR*; Higashi, 2000 ▶) *T*_min_ = 0.794, *T*_max_ = 0.862
53768 measured reflections
10948 independent reflections
10673 reflections with *I* > 2σ(*I*)
*R*_int_ = 0.025

#### Refinement

*R*[*F* ^2^ > 2σ(*F* ^2^)] = 0.041
*wR*(*F* ^2^) = 0.104
*S* = 1.07
10948 reflections
607 parameters
74 restraints
H-atom parameters constrained
Δρ_max_ = 1.16 e Å^−3^
Δρ_min_ = −1.02 e Å^−3^

### 

Data collection: *CrystalClear* (Rigaku, 2005 ▶); cell refinement: *CrystalClear*; data reduction: *CrystalClear*; program(s) used to solve structure: *SHELXS97* (Sheldrick, 2008 ▶); program(s) used to refine structure: *SHELXL2013* (Sheldrick, 2008 ▶); molecular graphics: *Mercury* (Macrae *et al.*, 2006 ▶) and *ORTEP-3 for Windows* (Farrugia, 2012 ▶); software used to prepare material for publication: *OLEX2* (Dolomanov *et al.*, 2009 ▶) and *publCIF* (Westrip, 2010 ▶).

## Supplementary Material

Crystal structure: contains datablock(s) I, global. DOI: 10.1107/S1600536813014712/hg5318sup1.cif

Structure factors: contains datablock(s) I. DOI: 10.1107/S1600536813014712/hg5318Isup2.hkl

Additional supplementary materials:  crystallographic information; 3D view; checkCIF report

## Figures and Tables

**Fig. 1.**
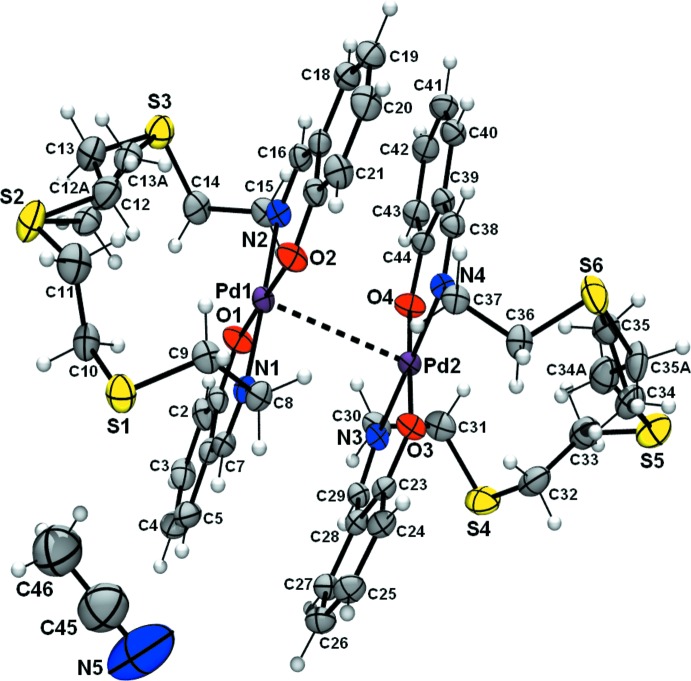
Asymmetric unit of the title compound, shown with 50% probability displacement ellipsoids. Labels for H-atoms, and C1, C6, C17, and C22 omitted for clarity.

**Fig. 2.**
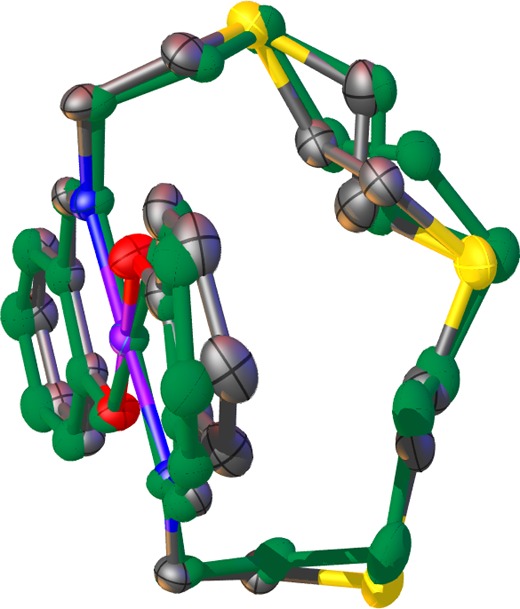
Overlay of both Pd complexes present in the asymmetric unit. The molecule containing Pd1 is green, while the molecule containing Pd2 has the atom colouring scheme Pd = purple, S = yellow, O = red, N = blue, C = grey. H-atoms omitted from this analysis.

**Fig. 3.**
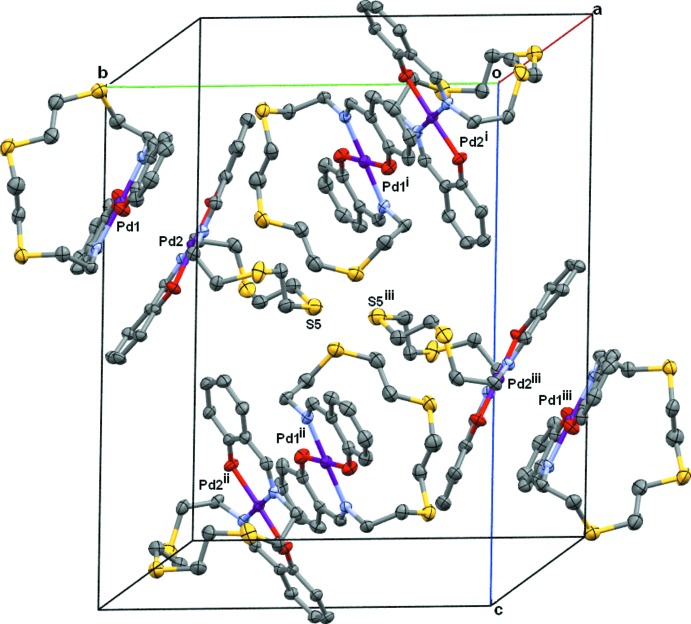
Packed unit cell for the title complex. H-atoms and minor disorder components omitted for clarity. Symmetry codes: (i) = 1 - x, -1/2 + y, 1/2 - z; (ii) = x, 1.5 - y, 1/2 + z; (iii) = 1 - x, 1 - y, 1 - z.

## supplementary crystallographic information

### Comment

The Pd coordination sphere in complexes containing salicylaldimine (sal) ligands significantly affects the catalytic properties of the compounds (Jin *et al.*, 2010). In particular, the tunability of the steric and electronic composition at the metal centre greatly influences catalytic performance.The heptadentate ligand present in the title complex has three different donor types, O, N and S, however, only the phenoxy O atoms and imine N atoms coordinate to the Pd^II^ cation, yielding a *trans*-N_2_O_2_ square planar geometry and two six-membered chelate rings. A search in the Cambridge Structural Database (CSD) *v.* 5.34 with Feb. 2013 update (Allen, 2002) for Pd complexes with molecules containing the noted three possible donor types (with coordination to no other elements allowed) yielded a total of 284 structures, wherein Pd was coordinated to just O and N donors in only 44 structures (and in order of decreasing frequency, the other structures had N & S = 99; only S = 53; O, N & S = 36; only N = 28; O & S = 24; and no examples where oxygen was the only donor.) Given the frequency in which S-coordination appears, it may be that it was not present as a donor in this structure due to the rigid framework established by the sal groups leading to pre-organization of the ligand for preferential coordination and formation of the six-membered chelate rings. A previously reported dinuclear Cu(II) complex containing the same ligand as the title complex, bis(µ_2_-2,2'-(5,8,11-trithia-2,14-diazapentadeca-1,14-diene-1,15-diyl)diphenolato)-di-copper(II), also exhibits coordination *via* only the phenoxy O atoms and imine N atoms, however, each copper site is square pyramidal, with axial coordination to a single phenoxy µ_2_-O donor found in the plane of the second copper (Lucas *et al.*, 2011*b*).

The asymmetric unit of the title complex contains two metal complex formula units (*Z*' = 2) and a single uncoordinated lattice solvent molecule of acetonitrile (Figure 1.) Part of the ligand chain (S—C—C—S) in each complex was disordered with two orientations, and refined occupancies that converged to 0.450 (10) and 0.550 (10) for the complex containing Pd1 (S2—C12—C13—S3 and S2A—C12A—C13A—S3A), and 0.789 (9) and 0.211 (9) for the complex containing Pd2 (S5—C34—C35—S6 and S5A—C34A—C35A—S6A.)

The two Pd-containing molecules in the asymmetric unit were overlayed (Figure 2; H-atoms omitted from this analysis), using *OLEX2* (Dolomanov *et al.*, 2009), giving an overall root-mean-square deviation of 0.292 Å. In this context, a comparison of the PdN_2_O_2_ mean planes to the terminal aromatic ring planes revealed significant deviations from planarity for the molecule containing Pd2 (plane-plane fold angles of 12.69 (8)° and 5.42 (9)° to the rings C23—C28 and C39—C44, respectively) compared with the near planar arrangement for the molecule containing Pd1 (1.19 (9)° and 4.32 (9)° to C1—C6 and C17—C22, respectively.)

While Pd^II^ is normally expected to exhibit a stable square planar geometry (16-electron rule), the presence of the occupied d*_z_*2 and the empty p*_z_* orbitals perpendicular to the coordination plane means that higher coordination numbers can be achieved, with the cation acting as a Lewis base, a Lewis acid, or both (Aullón & Alvarez, 1996). In the title complex, the shortest distance between Pd^II^ centres and possible intramolecular sulfur donors are 4.5864 (10) Å and 4.3964 (9) Å for Pd1—S3 and Pd2—S4 respectively, and therefore does not constitute an interaction at either Pd site. The Pd1—Pd2 separation, however, is 3.3776 (3) Å, while the PdN_2_O_2_ plane-plane fold angle is 1.62 (7) °. A search for close (sum of the van der Waals radii, 3.26 Å, + 0.4 Å) Pd—Pd separations in the CSD *v.* 5.34 with Feb. 2013 update (Allen, 2002) yielded 1137 observations with an average value of 3.2 (3). Examination of the packed unit cell revealed a short intermolecular S—S contact between S5 and the inversion related S5^iii^ (iii = 1 - *x*, 1 - *y*, 1 - *z*) measuring 3.663 (2) Å (Figure 3.)

### Experimental

All starting materials were obtained from the Aldrich Chemical Company and were used without further purification. Analyses were performed by Canadian Microanalytical Service Ltd.

*Preparation of the complex*, *[2,2'-(5,8,11-Trithia-2,14-diazapentadeca-1,14-diene-1,15-diyl)diphenolato]palladium(II) acetonitrile monosolvate*

Bis(acetonitrile)dichloropalladium(II) (0.259 g; 1.00 m mol) (Mathews *et al*., 2003) was dissolved in acetonitrile (50 ml) to give a yellow-orange solution. Likewise, 5,8,11-trithia-2,14-diazapentadeca-1,14-diene-1,15-diyl)diphenolate (0.449 g; 1.00 mmol) (Lucas *et al.* 2011*a*) was dissolved in acetonitrile (100 ml) to give a colourless solution. The solutions were mixed at room temperature, refluxed with stirring for 2 h, the volume reduced in a rotavap to ~75 ml and solvent then allowed to evaporate slowly at room temperature until orange crystals formed. These were removed by filtration at room temperature and dried in air to yield the product in 44% yield. Calc'd for C_44_H_52_N_4_O_4_Pd_2_S_6_^.^CH_3_CN: C 48.16; H 4.83; N 6.10; S 16.77. Found: C 48.08; H 4.72; N 6.49; S 16.25.

### Refinement

Hydrogen atoms were introduced into idealized positions and refined using the riding atom formalism (idealized Me refined as rotating group.) The applied constraints were: C_methine_—H_methine_ = 0.98 Å, C_methylene_—H_methylene_ = 0.97 Å, C_methyl_—H_methyl_ = 0.96 Å; *U*_iso_(H_methine_) = 1.2*U*_eq_(C_methine_), *U*_iso_(H_methylene_) = 1.2*U*_eq_(C_methylene_), *U*_iso_(H_methyl_) = 1.5*U*_eq_(C_methyl_).

Part of the ligand chain in each complex was disordered with two orientiations. For S2—C12—C13—S3 and S2A—C12A—C13A—S3A (and the pertinent H-atoms) the occupancies were constrained to equal to 1, and the respective occupancies resulted in 0.450 (10) and 0.550 (10). For S5—C34—C35—S6 and S5A—C34A—C35A—S6A (and the pertinent H-atoms) the occupancies were also constrained to equal to 1, and the respective occupancies resulted in 0.789 (9) and 0.211 (9). Similarity restraints (the command SAME and SIMU from *SHELXL-2013* by Sheldrick, 2008) were applied to {S2, C12, S2, C12A} {C13, S3, C13A, S3} {S5, C34, S6, C35} {S5, C34A, S6, C35A}. Distances were restrained (the command *DFIX* from *SHELXL-2013* by Sheldrick, 2008) for S5—C34A and S2—C12.

### Crystal data

**Table:**

[Pd(C_22_H_26_N_2_O_2_S_3_)]_2_·0.5C_2_H_3_N	*F*(000) = 2344
*M**_r_* = 1147.11	*D*_x_ = 1.586 Mg m^−^^3^
Monoclinic, *P*2_1_/*c*	Mo *K*α radiation, λ = 0.7107 Å
Hall symbol: -P 2ybc	Cell parameters from 22595 reflections
*a* = 14.7232 (5) Å	θ = 1.4–30.7°
*b* = 16.2151 (5) Å	µ = 1.06 mm^−^^1^
*c* = 20.9433 (8) Å	*T* = 153 K
β = 106.087 (1)°	Prism, yellow
*V* = 4804.2 (3) Å^3^	0.39 × 0.39 × 0.24 mm
*Z* = 4	

### Data collection

**Table:**

Rigaku Saturn70 diffractometer	10948 independent reflections
Radiation source: Sealed Tube	10673 reflections with *I* > 2σ(*I*)
Graphite monochromator	*R*_int_ = 0.025
Detector resolution: 28.5714 pixels mm^-1^	θ_max_ = 27.5°, θ_min_ = 2.9°
ω scans	*h* = −19→19
Absorption correction: numerical (*ABSCOR*; Higashi, 2000)	*k* = −20→20
*T*_min_ = 0.794, *T*_max_ = 0.862	*l* = −27→27
53768 measured reflections	

### Refinement

**Table:**

Refinement on *F*^2^	74 restraints
Least-squares matrix: full	Hydrogen site location: inferred from neighbouring sites
*R*[*F*^2^ > 2σ(*F*^2^)] = 0.041	H-atom parameters constrained
*wR*(*F*^2^) = 0.104	*w* = 1/[σ^2^(*F*_o_^2^) + (0.0515*P*)^2^ + 8.1878*P*] where *P* = (*F*_o_^2^ + 2*F*_c_^2^)/3
*S* = 1.07	(Δ/σ)_max_ = 0.004
10948 reflections	Δρ_max_ = 1.16 e Å^−^^3^
607 parameters	Δρ_min_ = −1.02 e Å^−^^3^

### Special details

**Table:**

Geometry. All e.s.d.'s (except the e.s.d. in the dihedral angle between two l.s. planes) are estimated using the full covariance matrix. The cell e.s.d.'s are taken into account individually in the estimation of e.s.d.'s in distances, angles and torsion angles; correlations between e.s.d.'s in cell parameters are only used when they are defined by crystal symmetry. An approximate (isotropic) treatment of cell e.s.d.'s is used for estimating e.s.d.'s involving l.s. planes.

### Fractional atomic coordinates and isotropic or equivalent isotropic displacement parameters (Å^2^)

**Table:**

	*x*	*y*	*z*	*U*_iso_*/*U*_eq_	Occ. (<1)
Pd1	0.24528 (2)	1.01728 (2)	0.25397 (2)	0.02431 (7)	
Pd2	0.27766 (2)	0.83920 (2)	0.34103 (2)	0.02261 (7)	
S1	0.27034 (7)	1.27406 (5)	0.35638 (5)	0.0436 (2)	
S2	0.18287 (8)	1.29247 (6)	0.13677 (5)	0.0542 (3)	
C12	0.2051 (8)	1.1866 (2)	0.1194 (5)	0.051 (3)	0.450 (10)
H12A	0.2739	1.1783	0.1268	0.061*	0.450 (10)
H12B	0.1839	1.1499	0.1502	0.061*	0.450 (10)
C12A	0.1402 (5)	1.1877 (4)	0.1129 (3)	0.0362 (16)	0.550 (10)
H12C	0.1328	1.1575	0.1522	0.043*	0.550 (10)
H12D	0.0778	1.1900	0.0794	0.043*	0.550 (10)
C13	0.1535 (7)	1.1637 (5)	0.0480 (5)	0.050 (3)	0.450 (10)
H13A	0.1790	1.1976	0.0177	0.060*	0.450 (10)
H13B	0.0858	1.1777	0.0397	0.060*	0.450 (10)
S3	0.16318 (7)	1.05643 (6)	0.02830 (5)	0.0455 (2)	
C13A	0.2104 (4)	1.1427 (4)	0.0841 (3)	0.0368 (17)	0.550 (10)
H13C	0.2623	1.1217	0.1214	0.044*	0.550 (10)
H13D	0.2383	1.1832	0.0595	0.044*	0.550 (10)
S4	0.09765 (8)	0.65616 (6)	0.40186 (6)	0.0517 (2)	
S5	0.38682 (8)	0.55470 (6)	0.47534 (5)	0.0515 (2)	
C34A	0.4263 (14)	0.6403 (8)	0.4351 (9)	0.050 (5)	0.211 (9)
H34A	0.4244	0.6250	0.3890	0.060*	0.211 (9)
H34B	0.3835	0.6880	0.4332	0.060*	0.211 (9)
S6	0.58158 (6)	0.74205 (6)	0.43321 (6)	0.0513 (2)	
C35A	0.5288 (13)	0.6647 (10)	0.4741 (10)	0.045 (5)	0.211 (9)
H35A	0.5689	0.6147	0.4811	0.054*	0.211 (9)
H35B	0.5283	0.6857	0.5184	0.054*	0.211 (9)
C34	0.4646 (4)	0.6428 (3)	0.4825 (2)	0.0435 (13)	0.789 (9)
H34C	0.4343	0.6914	0.4966	0.052*	0.789 (9)
H34D	0.5240	0.6315	0.5175	0.052*	0.789 (9)
C35	0.4880 (4)	0.6631 (3)	0.4188 (2)	0.0421 (13)	0.789 (9)
H35C	0.4307	0.6836	0.3857	0.051*	0.789 (9)
H35D	0.5095	0.6126	0.4007	0.051*	0.789 (9)
O1	0.10969 (14)	0.98736 (14)	0.22468 (11)	0.0348 (5)	
O2	0.38175 (14)	1.04476 (14)	0.28401 (11)	0.0336 (5)	
O3	0.32370 (14)	0.89742 (13)	0.42772 (10)	0.0294 (4)	
O4	0.23161 (14)	0.77858 (14)	0.25591 (10)	0.0322 (5)	
N1	0.23177 (17)	1.07243 (15)	0.33818 (12)	0.0262 (5)	
N2	0.25695 (17)	0.96422 (15)	0.16926 (12)	0.0274 (5)	
N3	0.14478 (16)	0.83256 (14)	0.35040 (12)	0.0249 (5)	
N4	0.41045 (16)	0.84643 (14)	0.33121 (12)	0.0256 (5)	
N5	0.0113 (7)	1.1934 (5)	0.4860 (4)	0.157 (3)	
C1	0.04531 (19)	1.00426 (18)	0.25485 (15)	0.0278 (6)	
C2	−0.0486 (2)	0.97597 (19)	0.22332 (18)	0.0343 (7)	
H2	−0.0610	0.9470	0.1824	0.041*	
C3	−0.1207 (2)	0.9903 (2)	0.25155 (18)	0.0383 (7)	
H3	−0.1825	0.9711	0.2299	0.046*	
C4	−0.1045 (2)	1.0326 (2)	0.31170 (18)	0.0419 (8)	
H4	−0.1550	1.0423	0.3308	0.050*	
C5	−0.0150 (2)	1.0602 (2)	0.34308 (17)	0.0378 (7)	
H5	−0.0042	1.0887	0.3841	0.045*	
C6	0.0612 (2)	1.04706 (18)	0.31557 (14)	0.0284 (6)	
C7	0.1526 (2)	1.07671 (18)	0.35308 (14)	0.0281 (6)	
H7	0.1548	1.1028	0.3941	0.034*	
C8	0.3150 (2)	1.10684 (19)	0.38663 (14)	0.0303 (6)	
H8A	0.3667	1.0658	0.3951	0.036*	
H8B	0.2993	1.1163	0.4290	0.036*	
C9	0.3503 (2)	1.1876 (2)	0.36453 (16)	0.0333 (6)	
H9A	0.3633	1.1784	0.3212	0.040*	
H9B	0.4109	1.2024	0.3970	0.040*	
C10	0.2018 (2)	1.2663 (2)	0.27020 (18)	0.0410 (7)	
H10A	0.1770	1.2094	0.2616	0.049*	
H10B	0.1470	1.3040	0.2626	0.049*	
C11	0.2570 (3)	1.2868 (3)	0.2211 (2)	0.0520 (9)	
H11A	0.3061	1.2442	0.2238	0.062*	
H11B	0.2894	1.3404	0.2333	0.062*	
C14	0.1084 (2)	0.9958 (2)	0.08026 (17)	0.0395 (7)	
H14A	0.0878	1.0334	0.1108	0.047*	
H14B	0.0513	0.9687	0.0516	0.047*	
C15	0.1726 (2)	0.9301 (2)	0.12107 (15)	0.0318 (6)	
H15A	0.1361	0.8974	0.1453	0.038*	
H15B	0.1930	0.8924	0.0906	0.038*	
C16	0.3354 (2)	0.95802 (18)	0.15369 (15)	0.0291 (6)	
H16	0.3319	0.9326	0.1122	0.035*	
C17	0.4278 (2)	0.98516 (18)	0.19181 (15)	0.0284 (6)	
C18	0.5036 (2)	0.9662 (2)	0.16524 (16)	0.0342 (6)	
H18	0.4909	0.9384	0.1238	0.041*	
C19	0.5947 (2)	0.9869 (2)	0.19757 (18)	0.0388 (7)	
H19	0.6449	0.9734	0.1791	0.047*	
C20	0.6127 (2)	1.0284 (2)	0.25844 (18)	0.0404 (7)	
H20	0.6757	1.0436	0.2811	0.048*	
C21	0.5405 (2)	1.0475 (2)	0.28593 (17)	0.0354 (7)	
H21	0.5546	1.0755	0.3273	0.042*	
C22	0.4460 (2)	1.02596 (18)	0.25362 (15)	0.0284 (6)	
C23	0.27123 (19)	0.93009 (17)	0.46249 (13)	0.0239 (5)	
C24	0.3174 (2)	0.97968 (18)	0.51772 (14)	0.0295 (6)	
H24	0.3842	0.9848	0.5296	0.035*	
C25	0.2674 (2)	1.02042 (19)	0.55435 (15)	0.0341 (7)	
H25	0.3001	1.0542	0.5905	0.041*	
C26	0.1689 (2)	1.0130 (2)	0.53941 (15)	0.0350 (7)	
H26	0.1344	1.0428	0.5640	0.042*	
C27	0.1235 (2)	0.96191 (19)	0.48844 (14)	0.0296 (6)	
H27	0.0572	0.9541	0.4796	0.035*	
C28	0.17248 (19)	0.92047 (17)	0.44875 (13)	0.0250 (5)	
C29	0.11687 (19)	0.86961 (17)	0.39603 (14)	0.0252 (5)	
H29	0.0523	0.8625	0.3947	0.030*	
C30	0.0742 (2)	0.7805 (2)	0.30476 (15)	0.0323 (6)	
H30A	0.0737	0.7932	0.2584	0.039*	
H30B	0.0107	0.7933	0.3097	0.039*	
C31	0.0947 (2)	0.6890 (2)	0.31811 (17)	0.0384 (7)	
H31A	0.1565	0.6761	0.3103	0.046*	
H31B	0.0458	0.6567	0.2859	0.046*	
C32	0.2233 (3)	0.6526 (2)	0.44326 (18)	0.0482 (9)	
H32A	0.2525	0.7051	0.4353	0.058*	
H32B	0.2325	0.6473	0.4917	0.058*	
C33	0.2735 (3)	0.5813 (2)	0.41969 (19)	0.0489 (9)	
H33A	0.2824	0.5958	0.3759	0.059*	
H33B	0.2321	0.5320	0.4133	0.059*	
C36	0.5182 (2)	0.83366 (19)	0.44539 (16)	0.0331 (6)	
H36A	0.4629	0.8169	0.4605	0.040*	
H36B	0.5600	0.8673	0.4811	0.040*	
C37	0.4843 (2)	0.88662 (19)	0.38354 (15)	0.0293 (6)	
H37A	0.4595	0.9393	0.3957	0.035*	
H37B	0.5386	0.8996	0.3661	0.035*	
C38	0.4345 (2)	0.81974 (18)	0.28014 (15)	0.0294 (6)	
H38	0.4987	0.8279	0.2810	0.035*	
C39	0.3762 (2)	0.77933 (18)	0.22229 (15)	0.0291 (6)	
C40	0.4179 (2)	0.76009 (19)	0.17117 (16)	0.0350 (7)	
H40	0.4828	0.7726	0.1771	0.042*	
C41	0.3672 (3)	0.7238 (2)	0.11291 (16)	0.0395 (7)	
H41	0.3964	0.7121	0.0787	0.047*	
C42	0.2726 (3)	0.7042 (2)	0.10454 (16)	0.0383 (7)	
H42	0.2373	0.6787	0.0645	0.046*	
C43	0.2293 (2)	0.7215 (2)	0.15391 (15)	0.0341 (6)	
H43	0.1650	0.7068	0.1477	0.041*	
C44	0.2795 (2)	0.76089 (17)	0.21343 (14)	0.0282 (6)	
C45	0.0247 (5)	1.2384 (5)	0.4458 (3)	0.098 (2)	
C46	0.0420 (5)	1.2925 (5)	0.3953 (4)	0.106 (2)	
H46A	−0.0058	1.2827	0.3530	0.159*	
H46B	0.1050	1.2813	0.3901	0.159*	
H46C	0.0386	1.3500	0.4088	0.159*	

### Atomic displacement parameters (Å^2^)

**Table:**

	*U*^11^	*U*^22^	*U*^33^	*U*^12^	*U*^13^	*U*^23^
Pd1	0.01968 (11)	0.02473 (12)	0.02705 (11)	0.00004 (7)	0.00403 (8)	−0.00088 (7)
Pd2	0.02143 (11)	0.02333 (11)	0.02199 (11)	−0.00194 (7)	0.00422 (8)	−0.00289 (7)
S1	0.0497 (5)	0.0313 (4)	0.0478 (5)	0.0008 (4)	0.0101 (4)	−0.0047 (3)
S2	0.0727 (7)	0.0348 (5)	0.0494 (5)	−0.0012 (4)	0.0076 (5)	0.0115 (4)
C12	0.066 (7)	0.040 (5)	0.050 (5)	−0.004 (4)	0.022 (4)	0.006 (4)
C12A	0.027 (3)	0.040 (3)	0.041 (3)	−0.005 (2)	0.009 (2)	0.008 (2)
C13	0.057 (6)	0.043 (5)	0.053 (5)	0.005 (4)	0.019 (5)	0.011 (4)
S3	0.0485 (5)	0.0461 (5)	0.0387 (4)	0.0025 (4)	0.0067 (4)	0.0033 (4)
C13A	0.027 (3)	0.038 (3)	0.045 (4)	−0.005 (2)	0.008 (3)	0.008 (3)
S4	0.0623 (6)	0.0443 (5)	0.0603 (6)	−0.0112 (4)	0.0365 (5)	0.0002 (4)
S5	0.0603 (6)	0.0481 (5)	0.0456 (5)	−0.0120 (5)	0.0138 (4)	−0.0002 (4)
C34A	0.062 (12)	0.039 (9)	0.046 (11)	0.004 (8)	0.010 (9)	0.001 (7)
S6	0.0334 (4)	0.0425 (5)	0.0810 (7)	0.0107 (4)	0.0208 (4)	0.0133 (5)
C35A	0.042 (9)	0.027 (7)	0.063 (12)	0.004 (6)	0.009 (8)	0.018 (7)
C34	0.050 (3)	0.044 (3)	0.033 (3)	−0.001 (2)	0.006 (2)	0.0023 (18)
C35	0.056 (3)	0.031 (2)	0.042 (3)	0.0071 (19)	0.018 (2)	0.0006 (17)
O1	0.0215 (10)	0.0432 (13)	0.0395 (12)	−0.0027 (9)	0.0084 (9)	−0.0106 (9)
O2	0.0223 (9)	0.0429 (12)	0.0342 (11)	−0.0008 (9)	0.0054 (8)	−0.0087 (9)
O3	0.0228 (9)	0.0359 (11)	0.0279 (10)	−0.0019 (8)	0.0046 (8)	−0.0115 (8)
O4	0.0287 (10)	0.0407 (12)	0.0274 (10)	−0.0074 (9)	0.0082 (8)	−0.0132 (9)
N1	0.0259 (11)	0.0241 (11)	0.0274 (11)	−0.0022 (9)	0.0055 (9)	0.0017 (9)
N2	0.0255 (11)	0.0265 (12)	0.0286 (12)	−0.0005 (9)	0.0047 (9)	−0.0040 (9)
N3	0.0204 (10)	0.0265 (12)	0.0256 (11)	−0.0009 (9)	0.0027 (9)	−0.0039 (9)
N4	0.0218 (11)	0.0255 (12)	0.0284 (12)	−0.0027 (9)	0.0054 (9)	−0.0016 (9)
N5	0.255 (10)	0.130 (6)	0.092 (5)	−0.069 (6)	0.059 (6)	−0.005 (4)
C1	0.0215 (13)	0.0242 (13)	0.0379 (15)	0.0013 (10)	0.0088 (11)	0.0054 (11)
C2	0.0245 (14)	0.0280 (15)	0.0487 (18)	0.0015 (11)	0.0070 (13)	0.0018 (13)
C3	0.0227 (14)	0.0371 (17)	0.054 (2)	−0.0008 (12)	0.0092 (13)	0.0097 (14)
C4	0.0293 (15)	0.054 (2)	0.0468 (19)	0.0048 (14)	0.0175 (14)	0.0135 (16)
C5	0.0361 (16)	0.0458 (19)	0.0342 (16)	0.0047 (14)	0.0142 (13)	0.0072 (13)
C6	0.0267 (13)	0.0269 (14)	0.0314 (14)	0.0029 (11)	0.0077 (11)	0.0091 (11)
C7	0.0316 (14)	0.0274 (14)	0.0253 (13)	0.0039 (11)	0.0080 (11)	0.0038 (10)
C8	0.0296 (14)	0.0325 (15)	0.0251 (13)	−0.0023 (12)	0.0015 (11)	−0.0001 (11)
C9	0.0247 (13)	0.0341 (16)	0.0389 (16)	−0.0034 (12)	0.0052 (12)	−0.0020 (12)
C10	0.0365 (17)	0.0332 (16)	0.0494 (19)	0.0033 (13)	0.0055 (14)	0.0065 (14)
C11	0.047 (2)	0.051 (2)	0.054 (2)	−0.0018 (17)	0.0072 (17)	0.0011 (18)
C14	0.0289 (15)	0.0462 (19)	0.0375 (17)	0.0015 (14)	−0.0009 (13)	−0.0011 (14)
C15	0.0260 (13)	0.0352 (16)	0.0309 (14)	−0.0028 (12)	0.0026 (11)	−0.0091 (12)
C16	0.0263 (13)	0.0304 (14)	0.0291 (14)	0.0042 (11)	0.0051 (11)	0.0005 (11)
C17	0.0267 (14)	0.0263 (14)	0.0315 (14)	0.0026 (11)	0.0071 (11)	0.0045 (11)
C18	0.0307 (15)	0.0395 (17)	0.0335 (15)	0.0036 (13)	0.0104 (12)	0.0038 (13)
C19	0.0258 (14)	0.049 (2)	0.0436 (18)	0.0034 (13)	0.0133 (13)	0.0081 (14)
C20	0.0240 (14)	0.0446 (19)	0.050 (2)	−0.0030 (13)	0.0056 (13)	0.0049 (15)
C21	0.0266 (14)	0.0373 (17)	0.0386 (16)	−0.0022 (12)	0.0032 (12)	−0.0024 (13)
C22	0.0238 (13)	0.0266 (14)	0.0348 (15)	0.0009 (11)	0.0079 (11)	0.0020 (11)
C23	0.0281 (13)	0.0231 (12)	0.0204 (12)	−0.0037 (10)	0.0066 (10)	0.0006 (9)
C24	0.0323 (14)	0.0304 (15)	0.0243 (13)	−0.0058 (12)	0.0055 (11)	−0.0019 (11)
C25	0.0423 (17)	0.0348 (16)	0.0233 (13)	−0.0067 (13)	0.0062 (12)	−0.0084 (11)
C26	0.0399 (17)	0.0380 (17)	0.0288 (15)	−0.0011 (13)	0.0124 (13)	−0.0048 (12)
C27	0.0287 (14)	0.0326 (15)	0.0279 (14)	−0.0005 (12)	0.0086 (11)	−0.0012 (11)
C28	0.0272 (13)	0.0248 (13)	0.0220 (12)	−0.0016 (11)	0.0051 (10)	0.0016 (10)
C29	0.0233 (12)	0.0249 (13)	0.0271 (13)	0.0000 (10)	0.0064 (10)	−0.0007 (10)
C30	0.0217 (13)	0.0388 (16)	0.0336 (15)	−0.0039 (12)	0.0033 (11)	−0.0122 (12)
C31	0.0378 (16)	0.0346 (16)	0.0431 (18)	−0.0115 (14)	0.0116 (14)	−0.0127 (13)
C32	0.073 (3)	0.0411 (19)	0.0332 (17)	−0.0045 (18)	0.0187 (17)	0.0019 (14)
C33	0.067 (2)	0.0416 (19)	0.0387 (18)	0.0001 (18)	0.0150 (17)	0.0001 (15)
C36	0.0272 (14)	0.0305 (15)	0.0367 (16)	−0.0024 (12)	0.0006 (12)	0.0006 (12)
C37	0.0228 (13)	0.0300 (14)	0.0336 (15)	−0.0059 (11)	0.0055 (11)	−0.0010 (11)
C38	0.0257 (13)	0.0297 (14)	0.0341 (15)	0.0002 (11)	0.0107 (11)	0.0007 (11)
C39	0.0323 (14)	0.0251 (13)	0.0311 (14)	0.0018 (11)	0.0106 (11)	−0.0008 (11)
C40	0.0409 (17)	0.0301 (15)	0.0384 (16)	0.0037 (13)	0.0184 (14)	−0.0006 (12)
C41	0.054 (2)	0.0360 (17)	0.0338 (16)	0.0035 (15)	0.0206 (15)	−0.0030 (13)
C42	0.0536 (19)	0.0321 (16)	0.0283 (15)	0.0028 (14)	0.0097 (14)	−0.0062 (12)
C43	0.0391 (16)	0.0304 (15)	0.0315 (15)	−0.0026 (13)	0.0074 (13)	−0.0073 (12)
C44	0.0357 (15)	0.0253 (13)	0.0236 (13)	0.0021 (11)	0.0083 (11)	−0.0015 (10)
C45	0.117 (5)	0.095 (5)	0.080 (4)	−0.011 (4)	0.024 (4)	−0.008 (4)
C46	0.105 (5)	0.109 (5)	0.110 (5)	0.001 (4)	0.038 (4)	0.012 (4)

### Geometric parameters (Å, º)

**Table:**

Pd1—O1	1.980 (2)	C8—H8B	0.9900
Pd1—O2	1.983 (2)	C8—C9	1.528 (4)
Pd1—N1	2.036 (2)	C9—H9A	0.9900
Pd1—N2	2.021 (2)	C9—H9B	0.9900
Pd2—O3	1.9913 (19)	C10—H10A	0.9900
Pd2—O4	1.9836 (19)	C10—H10B	0.9900
Pd2—N3	2.022 (2)	C10—C11	1.515 (5)
Pd2—N4	2.025 (2)	C11—H11A	0.9900
S1—C9	1.808 (3)	C11—H11B	0.9900
S1—C10	1.812 (4)	C14—H14A	0.9900
S2—C12	1.804 (2)	C14—H14B	0.9900
S2—C12A	1.833 (6)	C14—C15	1.519 (4)
S2—C11	1.802 (4)	C15—H15A	0.9900
C12—H12A	0.9900	C15—H15B	0.9900
C12—H12B	0.9900	C16—H16	0.9500
C12—C13	1.522 (14)	C16—C17	1.443 (4)
C12A—H12C	0.9900	C17—C18	1.411 (4)
C12A—H12D	0.9900	C17—C22	1.412 (4)
C12A—C13A	1.519 (9)	C18—H18	0.9500
C13—H13A	0.9900	C18—C19	1.367 (4)
C13—H13B	0.9900	C19—H19	0.9500
C13—S3	1.803 (9)	C19—C20	1.400 (5)
S3—C13A	1.832 (7)	C20—H20	0.9500
S3—C14	1.813 (4)	C20—C21	1.377 (5)
C13A—H13C	0.9900	C21—H21	0.9500
C13A—H13D	0.9900	C21—C22	1.413 (4)
S4—C31	1.822 (4)	C23—C24	1.418 (4)
S4—C32	1.813 (5)	C23—C28	1.410 (4)
S5—C34A	1.802 (2)	C24—H24	0.9500
S5—C34	1.811 (5)	C24—C25	1.371 (4)
S5—C33	1.803 (4)	C25—H25	0.9500
C34A—H34A	0.9900	C25—C26	1.401 (5)
C34A—H34B	0.9900	C26—H26	0.9500
C34A—C35A	1.55 (3)	C26—C27	1.371 (4)
S6—C35A	1.810 (17)	C27—H27	0.9500
S6—C35	1.843 (5)	C27—C28	1.413 (4)
S6—C36	1.809 (3)	C28—C29	1.438 (4)
C35A—H35A	0.9900	C29—H29	0.9500
C35A—H35B	0.9900	C30—H30A	0.9900
C34—H34C	0.9900	C30—H30B	0.9900
C34—H34D	0.9900	C30—C31	1.524 (5)
C34—C35	1.504 (7)	C31—H31A	0.9900
C35—H35C	0.9900	C31—H31B	0.9900
C35—H35D	0.9900	C32—H32A	0.9900
O1—C1	1.306 (4)	C32—H32B	0.9900
O2—C22	1.313 (4)	C32—C33	1.526 (5)
O3—C23	1.311 (3)	C33—H33A	0.9900
O4—C44	1.311 (3)	C33—H33B	0.9900
N1—C7	1.290 (4)	C36—H36A	0.9900
N1—C8	1.468 (4)	C36—H36B	0.9900
N2—C15	1.474 (4)	C36—C37	1.519 (4)
N2—C16	1.288 (4)	C37—H37A	0.9900
N3—C29	1.289 (4)	C37—H37B	0.9900
N3—C30	1.468 (3)	C38—H38	0.9500
N4—C37	1.465 (3)	C38—C39	1.435 (4)
N4—C38	1.291 (4)	C39—C40	1.409 (4)
N5—C45	1.170 (9)	C39—C44	1.415 (4)
C1—C2	1.432 (4)	C40—H40	0.9500
C1—C6	1.410 (4)	C40—C41	1.374 (5)
C2—H2	0.9500	C41—H41	0.9500
C2—C3	1.370 (5)	C41—C42	1.391 (5)
C3—H3	0.9500	C42—H42	0.9500
C3—C4	1.396 (5)	C42—C43	1.385 (4)
C4—H4	0.9500	C43—H43	0.9500
C4—C5	1.374 (5)	C43—C44	1.413 (4)
C5—H5	0.9500	C45—C46	1.450 (9)
C5—C6	1.413 (4)	C46—H46A	0.9800
C6—C7	1.439 (4)	C46—H46B	0.9800
C7—H7	0.9500	C46—H46C	0.9800
C8—H8A	0.9900		
			
O1—Pd1—O2	178.75 (10)	S2—C11—H11A	109.1
O1—Pd1—N1	92.51 (9)	S2—C11—H11B	109.1
O1—Pd1—N2	86.99 (9)	C10—C11—S2	112.5 (3)
O2—Pd1—N1	87.71 (9)	C10—C11—H11A	109.1
O2—Pd1—N2	92.81 (9)	C10—C11—H11B	109.1
N2—Pd1—N1	178.86 (10)	H11A—C11—H11B	107.8
O3—Pd2—N3	91.77 (9)	S3—C14—H14A	108.7
O3—Pd2—N4	88.33 (9)	S3—C14—H14B	108.7
O4—Pd2—O3	178.54 (9)	H14A—C14—H14B	107.6
O4—Pd2—N3	87.81 (9)	C15—C14—S3	114.1 (2)
O4—Pd2—N4	92.10 (9)	C15—C14—H14A	108.7
N3—Pd2—N4	179.63 (10)	C15—C14—H14B	108.7
C9—S1—C10	102.04 (16)	N2—C15—C14	113.4 (3)
C11—S2—C12	93.2 (4)	N2—C15—H15A	108.9
C11—S2—C12A	106.8 (2)	N2—C15—H15B	108.9
S2—C12—H12A	109.4	C14—C15—H15A	108.9
S2—C12—H12B	109.4	C14—C15—H15B	108.9
H12A—C12—H12B	108.0	H15A—C15—H15B	107.7
C13—C12—S2	111.2 (6)	N2—C16—H16	116.2
C13—C12—H12A	109.4	N2—C16—C17	127.6 (3)
C13—C12—H12B	109.4	C17—C16—H16	116.2
S2—C12A—H12C	109.8	C18—C17—C16	116.3 (3)
S2—C12A—H12D	109.8	C18—C17—C22	119.6 (3)
H12C—C12A—H12D	108.2	C22—C17—C16	124.1 (3)
C13A—C12A—S2	109.5 (4)	C17—C18—H18	119.1
C13A—C12A—H12C	109.8	C19—C18—C17	121.7 (3)
C13A—C12A—H12D	109.8	C19—C18—H18	119.1
C12—C13—H13A	108.7	C18—C19—H19	120.6
C12—C13—H13B	108.7	C18—C19—C20	118.8 (3)
C12—C13—S3	114.2 (6)	C20—C19—H19	120.6
H13A—C13—H13B	107.6	C19—C20—H20	119.5
S3—C13—H13A	108.7	C21—C20—C19	121.0 (3)
S3—C13—H13B	108.7	C21—C20—H20	119.5
C13—S3—C14	108.0 (3)	C20—C21—H21	119.4
C14—S3—C13A	100.6 (2)	C20—C21—C22	121.1 (3)
C12A—C13A—S3	116.1 (4)	C22—C21—H21	119.4
C12A—C13A—H13C	108.3	O2—C22—C17	125.1 (3)
C12A—C13A—H13D	108.3	O2—C22—C21	117.2 (3)
S3—C13A—H13C	108.3	C17—C22—C21	117.7 (3)
S3—C13A—H13D	108.3	O3—C23—C24	117.3 (2)
H13C—C13A—H13D	107.4	O3—C23—C28	125.1 (2)
C32—S4—C31	102.62 (16)	C28—C23—C24	117.6 (3)
C34A—S5—C33	82.8 (7)	C23—C24—H24	119.3
C33—S5—C34	108.4 (2)	C25—C24—C23	121.3 (3)
S5—C34A—H34A	109.7	C25—C24—H24	119.3
S5—C34A—H34B	109.7	C24—C25—H25	119.4
H34A—C34A—H34B	108.2	C24—C25—C26	121.1 (3)
C35A—C34A—S5	110.0 (11)	C26—C25—H25	119.4
C35A—C34A—H34A	109.7	C25—C26—H26	120.8
C35A—C34A—H34B	109.7	C27—C26—C25	118.5 (3)
C36—S6—C35A	101.3 (6)	C27—C26—H26	120.8
C36—S6—C35	101.68 (18)	C26—C27—H27	119.1
C34A—C35A—S6	114.2 (11)	C26—C27—C28	121.8 (3)
C34A—C35A—H35A	108.7	C28—C27—H27	119.1
C34A—C35A—H35B	108.7	C23—C28—C27	119.5 (3)
S6—C35A—H35A	108.7	C23—C28—C29	123.8 (3)
S6—C35A—H35B	108.7	C27—C28—C29	116.7 (3)
H35A—C35A—H35B	107.6	N3—C29—C28	127.3 (3)
S5—C34—H34C	108.9	N3—C29—H29	116.4
S5—C34—H34D	108.9	C28—C29—H29	116.4
H34C—C34—H34D	107.7	N3—C30—H30A	109.3
C35—C34—S5	113.2 (3)	N3—C30—H30B	109.3
C35—C34—H34C	108.9	N3—C30—C31	111.8 (2)
C35—C34—H34D	108.9	H30A—C30—H30B	107.9
S6—C35—H35C	109.5	C31—C30—H30A	109.3
S6—C35—H35D	109.5	C31—C30—H30B	109.3
C34—C35—S6	110.6 (3)	S4—C31—H31A	108.7
C34—C35—H35C	109.5	S4—C31—H31B	108.7
C34—C35—H35D	109.5	C30—C31—S4	114.3 (2)
H35C—C35—H35D	108.1	C30—C31—H31A	108.7
C1—O1—Pd1	127.13 (19)	C30—C31—H31B	108.7
C22—O2—Pd1	126.60 (19)	H31A—C31—H31B	107.6
C23—O3—Pd2	126.43 (17)	S4—C32—H32A	109.0
C44—O4—Pd2	127.16 (19)	S4—C32—H32B	109.0
C7—N1—Pd1	123.0 (2)	H32A—C32—H32B	107.8
C7—N1—C8	116.7 (2)	C33—C32—S4	113.0 (3)
C8—N1—Pd1	120.18 (19)	C33—C32—H32A	109.0
C15—N2—Pd1	120.00 (19)	C33—C32—H32B	109.0
C16—N2—Pd1	123.8 (2)	S5—C33—H33A	108.6
C16—N2—C15	116.2 (2)	S5—C33—H33B	108.6
C29—N3—Pd2	124.26 (19)	C32—C33—S5	114.6 (3)
C29—N3—C30	115.9 (2)	C32—C33—H33A	108.6
C30—N3—Pd2	119.78 (18)	C32—C33—H33B	108.6
C37—N4—Pd2	119.14 (18)	H33A—C33—H33B	107.6
C38—N4—Pd2	124.0 (2)	S6—C36—H36A	108.8
C38—N4—C37	116.8 (2)	S6—C36—H36B	108.8
O1—C1—C2	116.5 (3)	H36A—C36—H36B	107.7
O1—C1—C6	125.3 (3)	C37—C36—S6	113.9 (2)
C6—C1—C2	118.1 (3)	C37—C36—H36A	108.8
C1—C2—H2	119.6	C37—C36—H36B	108.8
C3—C2—C1	120.7 (3)	N4—C37—C36	112.8 (2)
C3—C2—H2	119.6	N4—C37—H37A	109.0
C2—C3—H3	119.5	N4—C37—H37B	109.0
C2—C3—C4	120.9 (3)	C36—C37—H37A	109.0
C4—C3—H3	119.5	C36—C37—H37B	109.0
C3—C4—H4	120.2	H37A—C37—H37B	107.8
C5—C4—C3	119.5 (3)	N4—C38—H38	116.0
C5—C4—H4	120.2	N4—C38—C39	128.0 (3)
C4—C5—H5	119.3	C39—C38—H38	116.0
C4—C5—C6	121.4 (3)	C40—C39—C38	117.2 (3)
C6—C5—H5	119.3	C40—C39—C44	119.2 (3)
C1—C6—C5	119.3 (3)	C44—C39—C38	123.5 (3)
C1—C6—C7	123.6 (3)	C39—C40—H40	119.2
C5—C6—C7	117.1 (3)	C41—C40—C39	121.7 (3)
N1—C7—C6	128.4 (3)	C41—C40—H40	119.2
N1—C7—H7	115.8	C40—C41—H41	120.4
C6—C7—H7	115.8	C40—C41—C42	119.2 (3)
N1—C8—H8A	108.8	C42—C41—H41	120.4
N1—C8—H8B	108.8	C41—C42—H42	119.6
N1—C8—C9	113.9 (2)	C43—C42—C41	120.7 (3)
H8A—C8—H8B	107.7	C43—C42—H42	119.6
C9—C8—H8A	108.8	C42—C43—H43	119.5
C9—C8—H8B	108.8	C42—C43—C44	120.9 (3)
S1—C9—H9A	108.5	C44—C43—H43	119.5
S1—C9—H9B	108.5	O4—C44—C39	125.1 (3)
C8—C9—S1	115.2 (2)	O4—C44—C43	116.7 (3)
C8—C9—H9A	108.5	C43—C44—C39	118.2 (3)
C8—C9—H9B	108.5	N5—C45—C46	178.7 (9)
H9A—C9—H9B	107.5	C45—C46—H46A	109.5
S1—C10—H10A	108.8	C45—C46—H46B	109.5
S1—C10—H10B	108.8	C45—C46—H46C	109.5
H10A—C10—H10B	107.7	H46A—C46—H46B	109.5
C11—C10—S1	114.0 (3)	H46A—C46—H46C	109.5
C11—C10—H10A	108.8	H46B—C46—H46C	109.5
C11—C10—H10B	108.8		
			
Pd1—O1—C1—C2	179.8 (2)	C2—C3—C4—C5	−0.1 (5)
Pd1—O1—C1—C6	−0.8 (4)	C3—C4—C5—C6	0.4 (5)
Pd1—O2—C22—C17	2.8 (4)	C4—C5—C6—C1	−0.4 (5)
Pd1—O2—C22—C21	−176.3 (2)	C4—C5—C6—C7	−178.6 (3)
Pd1—N1—C7—C6	−1.3 (4)	C5—C6—C7—N1	−179.6 (3)
Pd1—N1—C8—C9	74.4 (3)	C6—C1—C2—C3	0.0 (4)
Pd1—N2—C15—C14	75.9 (3)	C7—N1—C8—C9	−107.9 (3)
Pd1—N2—C16—C17	0.9 (4)	C8—N1—C7—C6	−179.0 (3)
Pd2—O3—C23—C24	−170.55 (19)	C9—S1—C10—C11	−69.8 (3)
Pd2—O3—C23—C28	8.7 (4)	C10—S1—C9—C8	−90.7 (3)
Pd2—O4—C44—C39	2.9 (4)	C11—S2—C12—C13	−179.2 (7)
Pd2—O4—C44—C43	−176.0 (2)	C11—S2—C12A—C13A	−88.3 (4)
Pd2—N3—C29—C28	−1.2 (4)	C14—S3—C13A—C12A	−53.6 (5)
Pd2—N3—C30—C31	70.9 (3)	C15—N2—C16—C17	−179.3 (3)
Pd2—N4—C37—C36	75.0 (3)	C16—N2—C15—C14	−103.9 (3)
Pd2—N4—C38—C39	−1.4 (4)	C16—C17—C18—C19	−178.4 (3)
S1—C10—C11—S2	−171.9 (2)	C16—C17—C22—O2	−0.4 (5)
S2—C12—C13—S3	−174.8 (5)	C16—C17—C22—C21	178.7 (3)
S2—C12A—C13A—S3	−156.5 (3)	C17—C18—C19—C20	−0.4 (5)
C12—S2—C12A—C13A	−22.9 (7)	C18—C17—C22—O2	−178.1 (3)
C12—S2—C11—C10	−94.3 (4)	C18—C17—C22—C21	1.0 (4)
C12—C13—S3—C13A	−18.9 (6)	C18—C19—C20—C21	0.7 (5)
C12—C13—S3—C14	62.5 (8)	C19—C20—C21—C22	−0.1 (5)
C12A—S2—C12—C13	61.4 (9)	C20—C21—C22—O2	178.5 (3)
C12A—S2—C11—C10	−67.6 (4)	C20—C21—C22—C17	−0.8 (5)
C13—S3—C13A—C12A	53.3 (7)	C22—C17—C18—C19	−0.5 (5)
C13—S3—C14—C15	−124.9 (4)	C23—C24—C25—C26	1.4 (5)
S3—C14—C15—N2	62.7 (3)	C23—C28—C29—N3	−7.3 (5)
C13A—S3—C14—C15	−92.2 (3)	C24—C23—C28—C27	2.0 (4)
S4—C32—C33—S5	−161.3 (2)	C24—C23—C28—C29	−177.6 (3)
S5—C34A—C35A—S6	173.0 (9)	C24—C25—C26—C27	2.0 (5)
S5—C34—C35—S6	−171.0 (2)	C25—C26—C27—C28	−3.5 (5)
C34A—S5—C34—C35	−27.6 (12)	C26—C27—C28—C23	1.5 (4)
C34A—S5—C33—C32	−81.6 (7)	C26—C27—C28—C29	−179.0 (3)
S6—C36—C37—N4	67.5 (3)	C27—C28—C29—N3	173.2 (3)
C35A—S6—C35—C34	20.5 (10)	C28—C23—C24—C25	−3.4 (4)
C35A—S6—C36—C37	−134.9 (7)	C29—N3—C30—C31	−106.9 (3)
C34—S5—C34A—C35A	24.5 (10)	C30—N3—C29—C28	176.6 (3)
C34—S5—C33—C32	−62.1 (3)	C31—S4—C32—C33	−71.1 (3)
C35—S6—C35A—C34A	−21.3 (9)	C32—S4—C31—C30	−99.9 (3)
C35—S6—C36—C37	−97.5 (3)	C33—S5—C34A—C35A	167.9 (14)
O1—C1—C2—C3	179.5 (3)	C33—S5—C34—C35	−66.2 (4)
O1—C1—C6—C5	−179.2 (3)	C36—S6—C35A—C34A	73.1 (14)
O1—C1—C6—C7	−1.1 (5)	C36—S6—C35—C34	−72.8 (3)
O3—C23—C24—C25	175.9 (3)	C37—N4—C38—C39	−179.3 (3)
O3—C23—C28—C27	−177.3 (3)	C38—N4—C37—C36	−107.1 (3)
O3—C23—C28—C29	3.2 (4)	C38—C39—C40—C41	−177.9 (3)
N1—C8—C9—S1	65.7 (3)	C38—C39—C44—O4	0.5 (5)
N2—C16—C17—C18	176.1 (3)	C38—C39—C44—C43	179.4 (3)
N2—C16—C17—C22	−1.7 (5)	C39—C40—C41—C42	−1.1 (5)
N3—C30—C31—S4	59.1 (3)	C40—C39—C44—O4	−177.2 (3)
N4—C38—C39—C40	176.5 (3)	C40—C39—C44—C43	1.7 (4)
N4—C38—C39—C44	−1.3 (5)	C40—C41—C42—C43	0.5 (5)
C1—C2—C3—C4	−0.1 (5)	C41—C42—C43—C44	1.2 (5)
C1—C6—C7—N1	2.4 (5)	C42—C43—C44—O4	176.7 (3)
C2—C1—C6—C5	0.2 (4)	C42—C43—C44—C39	−2.3 (5)
C2—C1—C6—C7	178.3 (3)	C44—C39—C40—C41	−0.1 (5)

## References

[bb1] Allen, F. H. (2002). *Acta Cryst.* B**58**, 380–388. 10.1107/s0108768102003890 12037359

[bb2] Aullón, G. & Alvarez, S. (1996). *Inorg. Chem.* **35**, 3137–3144. 10.1021/ic951643w 11666509

[bb3] Dolomanov, O. V., Bourhis, L. J., Gildea, R. J., Howard, J. A. K. & Puschmann, H. (2009). *J. Appl. Cryst.* **42**, 339–341.

[bb4] Farrugia, L. J. (2012). *J. Appl. Cryst.* **45**, 849–854.

[bb5] Higashi, T. (2000). *ABSCOR* Rigaku Corporation, Tokyo, Japan.

[bb6] Jin, Z., Qiu, L.-L., Li, Y.-Q., Hong, H.-B. & Fang, J.-X. (2010). *Organometallics*, **29**, 6578–6586.

[bb7] Lucas, C. R., Byrne, J. M. D., Collins, J. L., Dawe, L. N. & Miller, D. O. (2011*a*). *Can. J. Chem.* **89**, 1174–1189.

[bb8] Lucas, C. R., Byrne, J. M. D., Collins, J. L., Dawe, L. N. & Miller, D. O. (2011*b*). *Can. J. Chem.* **89**, 1190–1201.

[bb9] Macrae, C. F., Edgington, P. R., McCabe, P., Pidcock, E., Shields, G. P., Taylor, R., Towler, M. & van de Streek, J. (2006). *J. Appl. Cryst.* **39**, 453–457.

[bb10] Mathews, C. J., Smith, P. J. & Welton, T. (2003). *J. Mol. Catal. A Chem.* **206**, 77–82.

[bb11] Rigaku (2005). *Rigaku/AFC Diffractometer Control Software* Rigaku Corporation, Tokyo, Japan.

[bb12] Sheldrick, G. M. (2008). *Acta Cryst.* A**64**, 112–122. 10.1107/S0108767307043930 18156677

[bb13] Westrip, S. P. (2010). *J. Appl. Cryst.* **43**, 920–925.

